# PCR melting profile as a tool for outbreak studies of *Salmonella enterica* in chickens

**DOI:** 10.1186/s12917-015-0451-4

**Published:** 2015-06-23

**Authors:** Anna Zaczek, Arkadiusz Wojtasik, Radosław Izdebski, Elzbieta Gorecka, Ewelina A. Wojcik, Tomasz Nowak, Piotr Kwiecinski, Jaroslaw Dziadek

**Affiliations:** Department of Biochemistry and Cell Biology, University of Rzeszów, Rejtana 16c, 35-959 Rzeszów, Poland; Proteon Pharmaceuticals S.A., Tylna 3, 90-364 Łódź, Poland; Molecular Microbiology Department, National Medicines Institute, Chełmska 30/34, 00-725 Warsaw, Poland; Vet- Lab Brudzew, Turkowska 58C, 62-720 Brudzew, Poland; Institute of Medical Biology, Polish Academy of Science, Lodowa 106, 93-232 Łódź, Poland

**Keywords:** PCR MP, *Salmonella* differentiation, Infection outbreak monitoring

## Abstract

**Background:**

Salmonellosis is of great economic concern in all phases of the poultry industry, from production to marketing, leading to severe economic losses. Monitoring the source of the bacterial contamination has fundamental importance in the spreading of salmonellosis.

**Results:**

We applied a ligation-mediated PCR method, PCR MP (PCR melting profile), to type *S. enterica* ssp. *enterica* ser. Enteritidis (56 strains) and 43 control strains classified to other serovars isolated from poultry. We demonstrated the PCR MP potential for salmonellosis spreading monitoring. Our rapid test presents higher discriminatory power (0.939 vs. 0.608) compared to current molecular subtyping tool such as pulsed-field gel electrophoresis (PFGE), which ineffectiveness underlies the high degree of clonality of *S*. Enteritidis.

**Conclusions:**

PCR MP was found to be a highly discriminating, sensitive and specific method that could be a valuable molecular tool, particularly for analyzing epidemiological links of limited number of *S. enterica* ser*.* Enteritidis strains.

## Background

According to the most recent nomenclature adopted by the Centers for Disease Control (CDC), the genus *Salmonella* contains only two species, *Salmonella enterica* and *Salmonella bongori. S. enterica* is subdivided into six subspecies designated as follows: enterica (I), salamae (II), arizonae (IIIa), diarizonae (IIIb), houtenae (IV), and indica (VI), while *S. bongori* is not divided into subspecies. Both *Salmonella* species and subspecies are serotyped for further identification [1].

*Salmonella enterica* is a major cause of invasive infections and represents an important human and animal pathogen worldwide.

Non-typhoidal serovars of *S. enterica* can infect a broad range of domestic animals and cause different symptoms, ranging from gastroenteritis to death [2]. Some of these serovars, such as *S.* Typhimurium and *S.* Enteritidis, can infect animals and humans [3]. *Salmonella* outbreaks in humans are often associated with poultry and poultry products, which are considered reservoirs from which *Salmonella* is passed through the food chain and ultimately transmitted to humans [4]. Other serovars, such as *S.* Gallinarum and *S.* Pullorum in poultry, are host specific, infecting a single species and generally causing severe, typhoid-like symptoms leading to death [5]. In chickens, enteric disease caused by *S. enterica* is an important cause of mortality and morbidity. Monitoring of these bacteria, which may be associated with foodborne diseases in humans, is one of the great objectives of the poultry industry, since salmonellosis leads to severe economic losses.

An important challenge for the eradication of *Salmonella* is the development and implementation of rapid and affordable methods for the detection and characterization of this pathogen.

Although phenotyping approaches, such as serotyping or phage typing, are still commonly used in the investigation of *Salmonella* infections worldwide, these methods are most useful as preliminary tools for *Salmonella* classification. More efficient and precise molecular subtyping methods are needed to relate disease-causing pathogens to their probable sources and determine whether isolates from multiple, even widely dispersed, cases of salmonellosis are related [6].

Currently, pulsed-field gel electrophoresis (PFGE) is considered as the standard typing method for *Salmonella* outbreak investigations suitable for examining epidemiologically related strains. PFGE was adapted to *Salmonella* in the 1990s and was shown to have the capacity to identify strains at the origin of an outbreak [7, 8]. However, PFGE often may not be able to differentiate highly clonal strains [9, 10]. Moreover, PFGE is a time consuming and highly laborious method which can be performed only in reference laboratories.

Recently, several genotyping methods, such as MLST (multilocus sequence typing based on housekeeping genes), SNP, MLVA (multiple-locus VNTR analysis), MAPLT (multiple amplification of phage locus typing) and WGS (whole-genome sequencing) using NGS methods (next-generation sequencing) to identify subtypes by whole-genome comparisons, have been applied to analyze *Salmonella* strains [6, 11]. MLST appeared to be valuable for differentiating the major sublineages of *Salmonella*, so the molecular typing of *Salmonella* has been often performed based on variants of MLST [12–15]. WGS data are able to provide more accurate phylogenetic relationship than the small sets of genes used in MLST [11]. More recently, investigations of outbreaks are often based on MLVA, which generates reproducible results suitable for sharing between laboratories using the same standardized techniques [16–18]. MLVA appears to be as informative as WGS to determine the true underlying genetic relationships within *S.* Typhimurium [18]. However all of this methods require specialized equipment and analyses which are too expensive from the farmers point of view.

An interesting alternative to the methods mentioned above is the PCR melting profile (PCR MP) technique based on ligation-mediated PCR (LM-PCR), which has been useful in epidemiological analyses of a number of organisms. This technique was developed by Masny and Plucienniczak [19] and modified by Krawczyk et al. [20] for bacterial strain differentiation. To date, the PCR MP method has been successfully used for analyses of different strains of bacteria, such as *Escherichia coli, Klebsiella pneumoniae, Staphylococcus aureus, Enterococcus faecium, Clostridium difficile, Pseudomonas aeruginosa, Propionibacterium acnes*, as well as dermatophytes and yeast (*Candida*) [21, 22].

In this study, we adapted PCR MP to *Salmonella* for the first time. We found PCR MP useful in respect to discriminate closely related strains isolated at the same farm (different stables) or different farms served by the same hatchery. The utility of the PCR MP approach was evaluated by comparison of the results with data obtained using the PFGE method.

## Methods

### Bacterial strains

Ninety-nine *S. enterica* ssp. *enterica* strains were used in this study. In total, 56 strains of *S. enterica* ssp. *enterica ser.* Enteritidis (*S.* Enteritidis), 6 strains of *S.* Virchow, 1 strain of *S.* Senftenberg, 10 strains of *S.* Typhimurium, 5 strains of *S.* Infantis, 2 strains of *S.* Hadar, and 5 strains of *S.* Mbandaka were isolated from chickens and environmental samples by a veterinary diagnostic laboratory according to a National program for the control of certain Salmonella serotypes (Vet-Lab Brudzew, central Poland). All procedures were approved by Polish Centre for Accreditation on 18 July 2008 (Accreditation No. AB 924) and by the Chief Veterinary Officer Decision No. GIWhig-5120-23/08 on 24 October 2008. All strains were classified according to their growth requirements, colony morphology and biochemical characteristics and serotypes using ISO 6579:2002 (E) (Microbiology of food and animal feeding stuffs - Horizontal method for the detection of *Salmonella* spp.)

The *S.* Berta, *S.* Colindale, *S.* Derby, *S.* Enteritidis, *S.* Heidelberg, *S.* Moscow, and *S.* Virchow control strains were obtained from the museum collection of National Veterinary Research Institute PIWet Pulawy (animal origin), and the *S.* Brandenburg, *S.* Enteritidis (2), *S.* Hadar, *S.* Paratyphi, and *S.* Typhimurium (2) control strains were obtained from the Sanepid sanitary-epidemiological station in Lodz. All strains were cultured in LB medium (10 g/L trypton, 5 g/L yeast extract, 10 g/L NaCl, pH 7.0) for 20 h at 37°C.

### DNA extraction

The genomic DNA was extracted from each strain after overnight culture on LB agar using the Genomic Mini system (A&A Biotechnology). The DNA in the samples was quantified using an ND-1000 Spectrophotometer (NanoDrop Technologies Inc., USA).

### PCR MP

The PCR MP procedure, which was initially developed for the differentiation of *Escherichia coli* [20], was based on the digestion of genomic DNA with restriction enzymes and the ligation of the obtained DNA restriction fragments with an oligonucleotide adaptor followed by PCR amplification with a reduction of the denaturation temperature during each cycle.

The PCR MP procedure was optimized for *Salmonella* spp. In this study, we digested genomic DNA (about 0.5 μg) by incubating a mixture containing 10 U of HindIII (1.0 μl) (Fast Digest; Fermentas, Lithuania) and 2.0 μl of reaction buffer in a total volume of 20 μl at 37 °C for 15 min. Next, the digested genomic DNA was ligated to the adaptor (1 μM) using 0.5 U of T4 ligase and 2.5 μl of 1x ligation buffer (Fermentas) in a total volume of 25 μl for 1 h at 25 °C. The adaptor was prepared by mixing equimolar amounts of two oligonucleotides: pcr/mp-oligo-ligCTCACTCTCACCAACGTCGAC, and oli-pom-HindIII AGCTGTCGACGTTGG (Eurogentec, Belgium) dissolved in 100 μl water to a final concentration of 10 μM and incubated for 2 min at 60 °C. After ligation of the digested genomic DNA with the adaptor, the mixture was heated in a thermo-block at 70 °C for 10 min and then cooled. A total of 1 μl of this mixture was amplified by PCR (Verity thermocycler; Applied Biosystems, USA) in a reaction mixture consisting of 20 pmol of primer (pcr/mp-starter-Hind: CTCACTCTCACCAACGTCGACAGCTT), 1x PCR buffer Shark (200 μM Tris–HCl pH 8.8, 100 mM KCl, 100 mM (NH_4_)_2_SO_4_, 1 % Triton X-100, DNA Gdańsk), 1.5 mM MgCl_2_, 0.8 mM nucleoside triphosphates, and 1 U of *Pwo* polymerase *Hypernova* (DNA Gdańsk II, Poland) in a total volume of 25 μl. The denaturation temperature was determined during the optimization experiments for two genetically unrelated *S. enterica* strains using a gradient thermal cycler (Biometra, T-Gradient) with a gradient range of 83.5 – 88.5 °C for the denaturation step. The PCRs were performed as follows: 7 min at 72 °C; an initial denaturation step for 90 s over a gradient of 83.5 – 88.5 °C; 22 cycles of denaturation for 1 min at a gradient of 83.5 – 88.5 °C followed by annealing and elongation at 72 °C for 2 min 15 s; and a final elongation step at 72 °C for 5 min. For all isolates of *Salmonella*, the PCRs were performed at least three times as described above using the established optimal temperature of 85.7 °C.

Each PCR product (8 μl) was run on a 6 % polyacrylamide gel (AppliChem, Germany), and the amplification patterns were determined by examination on ethidium bromide (0.7 %)-stained gels illuminated by UV light (Alpha Innotech, Fc8800). The amplicon sizes were determined by comparing the bands with a 100-bp DNA mass ladder (Fermentas). Electrophoresis images were collected. The total procedure of PCR-MP was completed within 5 h.

Eight independent PCR MP reactions were conducted for *S.* Enteritidis 571 and *S.* Typhimurium 1751 to confirm the repeatability of the method.

### PFGE

Pulsed-field gel electrophoresis (PFGE) was performed as described [23] with the use of the XbaI restriction enzyme (Fermentas, Vilnius, Lithuania). The PFGE types and subtypes were discerned visually according to the criteria by Tenover et al. [24].

### Data analysis

Epidemiological data were analyzed using the BioNumerics package (Version 6.01, Applied Maths, Sint-Martens-Latem, Belgium) based on images of PCR MP electrophoretic band patterns obtained for the entire collection of strains. Dendrograms were generated with BioNumerics software using the Dice similarity coefficient and clustering by the unweighted pair group method with arithmetic mean (UPGMA), with 1 % tolerance for differences in the band position. A cluster was defined here as all isolates sharing the same pattern. The Hunter-Gaston discriminatory index (HGDI) was calculated as described previously [25], and it was used to evaluate the discriminatory power of the typing methods.

## Results

### Population structure in PFGE genotyping

**Fig. 1:**
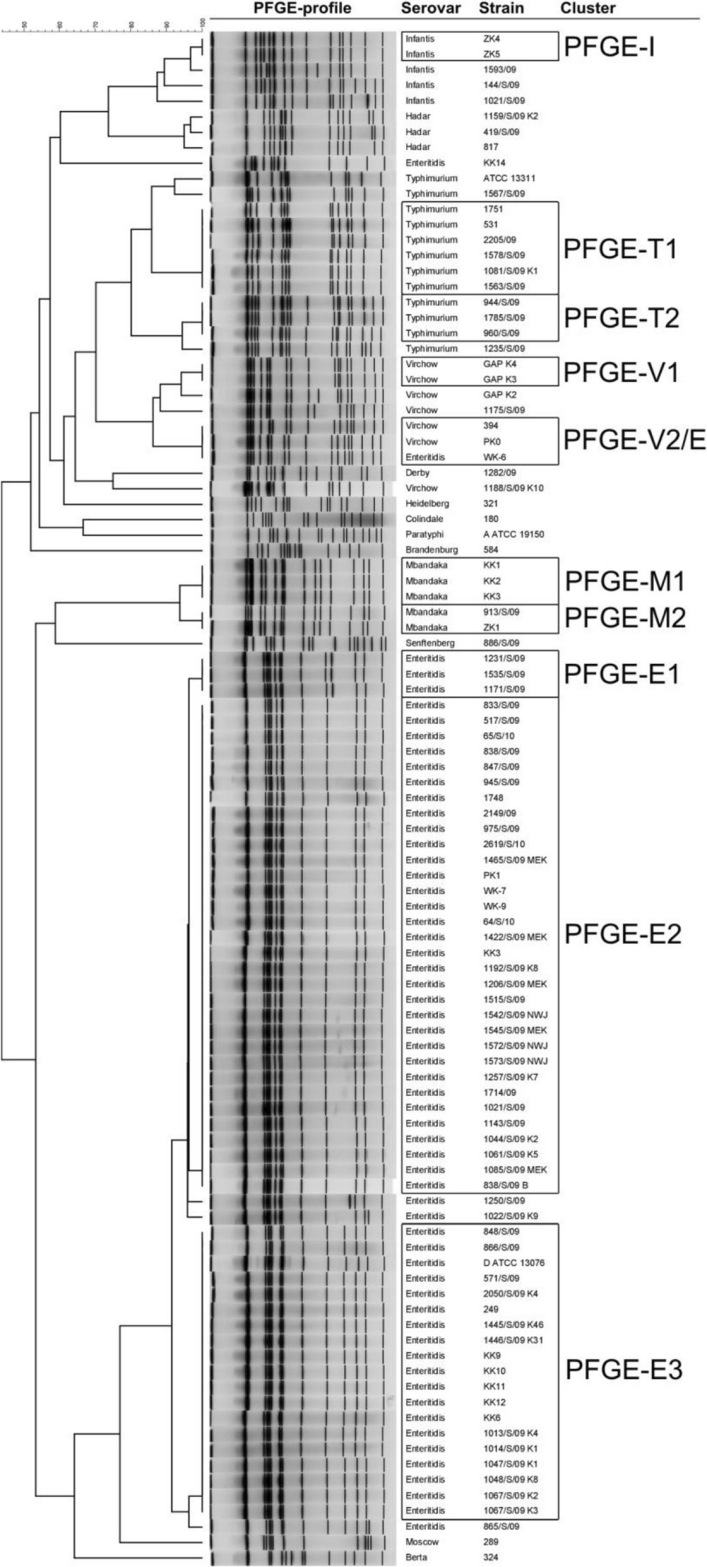
Differentiation of a collection of 99 *S. enterica* ssp. *enterica* strains by PFGE method. Genetic relationships among collection of *S. enterica* strains based on PFGE patterns. The serovars and strain numbers are indicated on the right of the band patterns. The similarity among strains, as a percentage, is indicated above the dendrogram. Strains grouped in clusters are framed. The tree was generated using the Dice correlation and unweighted pair group, as described in Methods

A collection of 99 *S. enterica subsp. enterica* strains was used for genotyping using the PFGE method. As can be clearly seen in figure (Fig. 1), it was possible to identify 10 clusters comprising approximately 75 % of the strains, whereas the remaining 25 patterns were unique and accordingly defined as singletons. Particularly, the most numerous serovar, *S.* Enteritidis, was divided into 1 small (PFGE-E1) and 2 large clusters (PFGE-E2 and PFGE-E3), which included 3, 32 and 19 strains, respectively. Only 5 patterns were recognized as unique; of these 5 strains, 3 displayed a high similarity to clustered strains, whereas the profiles of strains the KK14 and WK-6 were completely unique. The *S.* Typhimurium group (12 strains) was characterized by 3 singletons and 2 clusters (PFGE-T1 and PFGE-T2) with 6 and 3 strains included, respectively. For *S.* Virchow (7 strains), with the exception of 3 singletons, 2 clusters (PFGE-V1 and PFGE-V2/E) were identified. The second of these was surprisingly composed of 2 *S.* Virchow strains (394 and PK0) and, separately, strain WK-6 of the *S.* Enteritidis group. The serovar Mbandaka (5 strains) was distributed among 2 clusters (PFGE-M1 and PFGE-M2), and Infantis (5 strains) had 3 unique band profiles and 1 cluster (PFGE-I). Three *S.* Hadar strains and the remaining single representatives of the different serovars were categorized as singletons.

### Population structure by PCR MP genotyping

**Fig. 2:**
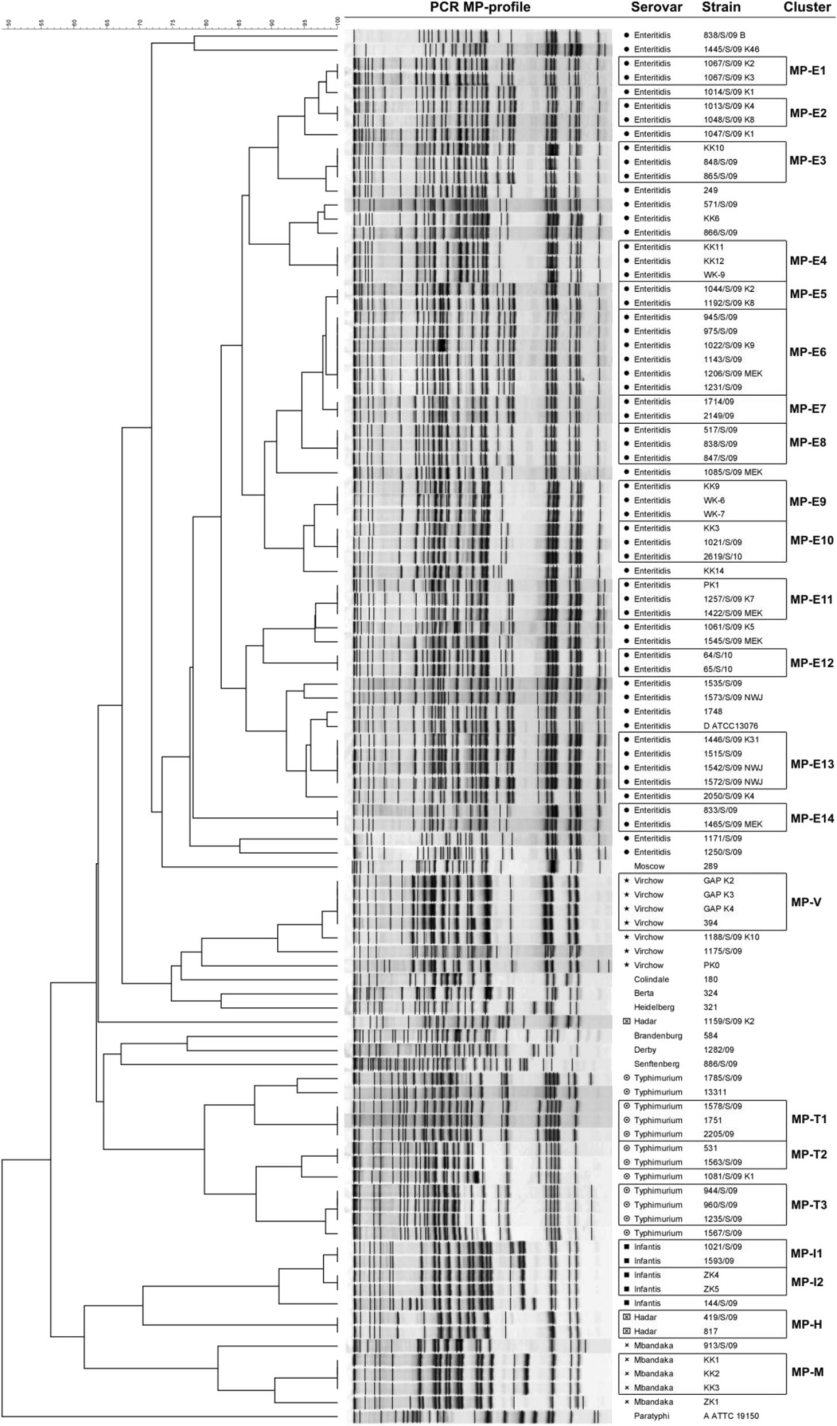
Differentiation of a collection of 99 *S. enterica* ssp. *enterica* strains by PCR MP method. Genetic relationships among collection of *S. enterica* strains based on PCR MP profiles. The serovars and strain numbers are indicated on the right of the band patterns. The similarity among strains, as a percentage, is indicated above the dendrogram. Strains grouped in clusters are framed. The tree was generated using the Dice correlation and unweighted pair group, as described in Methods

After the repeatability of the PCR MP method confirmation based on eight independent reactions conducted for *S.* Enteritidis 571 and *S.* Typhimurium 1751 all 99 of the analyzed isolates were typeable using the PCR MP approach. Based on the genotyping results, 38 unique patterns and 22 clusters containing low numbers of isolates comprised approximately 62 % of the strains (Fig. 2). In the case of the *S.* Enteritidis set, PCR MP differentiated up to 19 singletons and 14 small clusters (from MP-E1 to MP-E14), including strains in number of 2 to 6. In contrast to the PFGE, patterns that differed substantially from the *S.* Enteritidis group were not detected. Using the PCR MP method, it was possible to generate 3 clusters (from MP-T1 to MP-T3) for the *S.* Typhimurium strains, whereas 4 patterns of this serovar displayed unique profiles. Among the *S.* Virchow collection, 3 strains had different band profiles, and the 4 remaining strains clustered together (MP-V). In contrast to the PFGE results, no similarity was found in the profile between the *S.* Virchow strains (394 and PK0) and *S.* Enteritidis WK-6. A group of *S.* Mbandaka strains revealed the same cluster that was detected by PFGE (MP-M), whereas the profiles of the 2 remaining strains were found to be different. On the contrary, in the case of *S.* Hadar, two of three strains were clustered (MP-H) according to the PCR MP results. Strains belonging to the *S.* Infantis serovar were distributed among 2 clusters (MP-I1 and MP-I2), and one pattern appeared to be a singleton. As expected, all single representatives of different serovars were found to be singletons.

### Comparison of the strain discrimination potential of the PFGE and PCR MP methods regarding the *S.* Enteritidis serovar

The more detailed comparison of the two methods was implemented for the *S.* Enteritidis collection due to the number of strains in the analyzed population. The PCR MP typing approach displayed higher differentiating power than the reference method, reaching discriminatory index (HGDI) of 0.939 in comparison to 0.608. Additionally, the intrinsically lower numbers of bands in the PFGE patterns might contribute to the moderately lower resolution of this method compared with PCR MP genotyping. A cross-classification pattern for the clustered strains determined using both methods is shown to provide a more clear depiction of the relationship (Table 1). When taking into consideration two large PFGE-E clusters, it is clearly demonstrated that strains from both of these clusters were almost regularly distributed among the MP-E types as well as differentiated as singletons. However, it is worth mentioning that 3 of 5 singletons generated by PFGE were clustered with other strains identified using the PCR MP approach, and they could not be differentiated by PCR MP typing alone. This outcome strongly suggested that PFGE could serve as reasonable complementary approach during a detailed epidemiological analysis.

**Table 1:** Discrimination potential of the PFGE and PCR MP regarding 56 *S.* Enteritidis strains

		MP-E cluster															
		1	2	3	4	5	6	7	8	9	10	11	12	13	14	singleton	total
PFGE-E cluster	1						1									2	**3**
	2				1	2	4	2	2	1	3	3	2	3	2	7	**32**
	3	2	2	2	2				1	1				1		8	**19**
	singleton			1			1			1						2	**5**
	**total**	**2**	**2**	**3**	**3**	**2**	**6**	**2**	**3**	**3**	**3**	**3**	**2**	**4**	**2**	**19**	**59**

### Epidemiological links

All 99 analyzed *S. enterica* ssp. *enterica* strains were grouped into MP clusters, as shown above. Some clusters, such as MP-E1 and MP-E4, a portion of MP-E6, MP-E9, and MP-E12, and a portion of MP-E13, MP-V, MP-I2, and MP-M, were composed of strains isolated from poultry hatched in the same hatchery and bred at the same or similar time on farms belonging to the same farmer (Table 2). Other clusters, such as MP-E2, MP-E4, MP-E9, MP-E11, MP-T3, and MP-I1, originated from the same hatchery, which indicates that the hatchery was the source of *Salmonella* infection. It was also observed that the *Salmonella* strains infecting broiler chickens were isolated from coops belonging to the same farmer. In this case, the source of *Salmonella* infection was the farmer and not the hatchery (*S.* Mbandaka 913/S/09 and MP-M). Some isolates originating from chickens hatched in the same hatchery and bred by the same farmer did not group within the same clusters (MP-E1 and *S.* Enteritidis 1014/S/09 K1; MP-E4 and *S.* Enteritidis 1044/S/09 K2). In this case, the *Salmonella* strains were isolated from chickens bred in different batches or during different periods of time (Table 2).

**Table 2:** Epidemiological data of 99 *S. enterica* ssp. *enterica* strains used in study

Serovar	MP Cluster	Strain	Source: farm/isolation date	Epidemiological links			
				Farmer	Hatchery	Kind of poultry/Sample	Coexistence
Enteritidis		838/S/09 B	Konin/16.06.2009	F1	H1	(CL)	E
		1445/S/09 K46	Poddębice/25.08.2009	F1	H1	(CL)	L
	MP-E1	1067/S/09 K2	Brodnica/13.07.2009	F5	H4	(CL)	K
		1067/S/09 K3	Brodnica/13.07.2009	F5	H4	(CL)	K
		1014/S/09 K1	Brodnica/07.07.2009	F5	H4	(CL)	H
	MP-E2	1013/S/09 K4	Brodnica/07.07.2009	F5	H1	(CL)	H
		1048/S/09 K8	Konin/10.07.2009	F1	H1	(CL)	J
		1047/S/09 K1	Konin/10.07.2009	F1	H1	(CL)	J
	MP-E3	KK10	Konin/20.02.2009	F1	H1	(CL)	A
		848/S/09	Słupca/16.06.2009	F7	No data	(CB)	F
		865/S/09	Mińsk Mazowieck/18.06.2009	F8	H5	(BB)	G
		249	Control (PIWet Puławy)			(S)	
		571/S/09	Brodnica/06.05.2009	F5	H4	(CL)	
		KK6	Konin/20.02.2009	F1	H1	(CL)	A
		866/S/09	Mińsk Mazowieck/18.06.2009	F8	H5	(BB)	G
	MP-E4	KK11	Konin/20.02.2009	F1	H1	(CL)	A
		KK12	Konin/20.02.2009	F1	H1	(CL)	A
		WK-9	Poddębice/28.03.2009	F1	H1	(CL)	C
	MP-E5	1044/S/09 K2	Konin/10.07.2009	F1	H1	(CL)	J
		1192/S/09 K8	Poznań/25.07.2009	F13	No data	(CB)	
	MP-E6	945/S/09	Żyrardów/26.06.2009	F9	H6	(CB)	
		975/S/09	Łomża/30.06.2009	F10	No data	(CB)	
		1022/S/09 K9	Turek/08.07.2009	F6	H7	(CB)	I
		1143/S/09	Konin/21.07.2009	F1	H1	(CL)	
		1206/S/09 MEK	Konin/29.07.2009	F1	H1	(CL)	
		1231/S/09	No data	No data	No data		
	MP-E7	1714/09	Konin/24.09.2009	F16	No data	(CB)	
		2149/09	Siedlce/13.11.2009	F18	No data	(CB)	
	MP-E8	517/S/09	Piotrków Trybunalski/27.04.2009	F4	No data	(CB)	
		838/S/09	Konin/16.06.2009	F1	H1	(CL)	E
		847/S/09	Słupca/16.06.2009	F7	No data	(CB)	F
		1085/S/09 MEK	Słupca/14.07.2009	F7	H2	(CB)	
	MP-E9	KK9	Konin/20.02.2009	F1	H1	(CL)	A
		WK-6	Poddębice/28.03.2009	F1	H1	(CL)	C
		WK-7	Poddębice/28.03.2009	F1	H1	(CL)	C
	MP-E10	KK3	Konin/20.02.2009	F1	H1	(CL)	A
		1021/S/09	Turek/08.07.2009	F6	H7	(CB)	I
		2619/S/10	Słupca/17.07.2010	F7	H7	(CB)	
		KK14	Konin/20.02.2009	F1	H1	(CL)	A
	MP-E11	PK1	Krotoszyn/10.07.2009	F2	H2	(CB)	B
		1257/S/09 K7	Słupca/03.08.2009	F7	H2	(CB)	
		1422/S/09 MEK	Zduńska Wola/21.08.2009	F15	H2	(CB)	
		1061/S/09 K5	Słupca/12.07.2009	F7	No data	(CB)	
		1545/S/09 MEK	Słupca/08.09.2009	F7	H2	(CB)	M
	MP-E12	64/S/10	Siedlce/12.01.2010	F3	No data	(CB)	D
		65/S/10	Siedlce/12.01.2010	F3	No data	(CB)	D
		1535/S/09	Poznań/05.09.2009	F13	No data	(CB)	
		1573/S/09 NWJ	Słupca/11.09.2009	F7	H2	(CB)	N
		1748	Control (Sanepid)			(S)	
		D ATCC 13076	Control (Sanepid)			(S)	
	MP-E13	1446/S/09 K31	Poddębice/25.08.2009	F1	H1	(CL)	L
		1515/S/09	Mińsk Mazowiecki/03.09.2009	F8	H5	(BB)	
		1542/S/09 NWJ	Słupca/08.09.2009	F7	H2	(CB)	M
		1572/S/09 NWJ	Słupca/11.09.2009	F7	H2	(CB)	N
		2050/S/09 K4	Kalisz/30.10.2009	F17	No data	(CB)	
	MP-E14	833/S/09	Turek/15.06.2009	F6	H7	(CB)	
		1465/S/09 MEK	No data	No data	No data		
		1171/S/09	Łosice/22.07.2009	F12	No data	(BB)	
		1250/S/09	Ostrów Wlkp./03.08.2009	F14	No data	(CB)	
Moscow		289	Control (PIWet Puławy)			(S)	
Virchow	MP-V	GAP K2	Brodnica/03.11.2009	F5	H4	(CL)	O
		GAP K3	Brodnica/03.11.2009	F5	H4	(CL)	O
		GAP K4	Brodnica/03.11.2009	F5	H4	(CL)	O
		394	Control (PIWet Puławy)/2009			(S)	
		1188/S/09 K10	Konin/24.07.2009	F19	No data	(CB)	
		1175/S/09	Żuromin/23.07.2009	F23	No data	(CL)	
		PK0	Krotoszyn/10.07.2009	F2	H2	(CB)	B
Colindale		180	Control (PIWet Puławy)			(S)	
Berta		324	Control (PIWet Puławy)			(S)	
Heidelberg		321	Control (PIWet Puławy)			(S)	
Hadar		1159/S/09 K2	Łomża/22.07.2009	F10	No data	(CB)	
Brandenburg		584	Control (Sanepid)			(S)	
Derby		1282/09	Control (PIWet Puławy)			(S)	
Senftenberg		F23/S/09	Żuromin/19.06.2009		No data	(CL)	
Typhimurium		1785/S/09	Radziejów/01.10.2009	F29	No data	(CB)	
		ATCC 13311	Control (Sanepid)			(S)	
	MP-T1	1578/S/09	Kalisz/11.09.2009	F17	No data	(CB)	
		1751	Control (Sanepid)			(S)	
		2205/09	Węgrów/9.11.2009	F30	No data	(ES)	
	MP-T2	531	Nowy Tomyśl/29.04.2009	F24	No data	(CB)	
		1563/S/09	Żyrardów/10.09.2009	F27	No data	(CB)	
		1081/S/09 K1	Żuromin/14.07.2009	F23	No data	(CL)	
	MP-T3	944/S/09	Poddębice/26.06.2009	F25	H3	(G)	
		960/S/09	Konin/29.06.2009	F26	H3	(G)	
		1235/S/09	Poddębice/30.07.2009	F25	H3	(G)	
		1567/S/09	Kalisz/10.09.2009	F28	No data	(G)	
Infantis	MP-I1	1021/S/09	Turek/08.07.2009	F6	H7	(CB)	
		1593/09	Koło/14.09.2009	F21	H7	(CB)	
	MP-I2	ZK4	Konin/01.04.2009	F19	H7	(CB)	Q
		ZK5	Konin/01.04.2009	F19	H7	(CB)	Q
		144/S/09	Września/10.02.2009	F20	No data	(CB)	
Hadar	MP-H	419/S/09	Konin/06.04.2009	F16	H8	(ES)	
		817	Control (Sanepid)			(S)	
Mbandaka		913/S/09	Ostrów Wlkp./23.06.2009	F22	H4	(CB)	
	MP-M	KK1	Mława/11.03.2009	F31	H4	(CB)	P
		KK2	Mława/11.03.2009	F31	H4	(CB)	P
		KK3	Mława/11.03.2009	F31	H4	(CB)	P
		ZK1	Konin/01.04.2009	F19	No data	(CB)	Q
Paratyphi		A ATCC 19150	Control (Sanepid)			(S)	

Coexistence column indicates chickens having contact with each other (the same age, the same farm) marked by the same letter

Kind of poultry/sample column indicates: *CL* commercial layer, *CB* commercial broiler, *BB* broiler breeder, *G* goose, *S* control strain, *ES* environmental swabs

## Discussion

The gold standard of typing techniques might appear soon the sequencing of the whole genome of a pathogen, which has the highest discriminatory power [26–28]. However, for epidemiological purposes, methods with lower discriminatory power are sufficient for many diseases as far as public health is concerned. The investigation of outbreaks, and especially the routine control of *Salmonella* spread in the poultry industry by monitoring stable or hatchery contamination, requires rapid and inexpensive methods. An interesting alternative to the current set of methods, routinely used for typing of *Salmonella*, could be ligation-mediated PCR (LM-PCR), which has proven its usefulness in the epidemiological analysis of a number of bacterial species [19, 20, 29]. PCR MP, which controls a number of DNA restriction fragments to be amplified by decreasing the temperature of the denaturation step, was also successfully used [20]. Here, for the first time, we applied PCR MP for the molecular typing of *Salmonella* strains isolated from both chickens and environmental samples, and we compared the results to those of PFGE with the same collection. The most frequently detected representative of our collection was serovar Enteritidis with 56 strains. It was clearly demonstrated that the discriminatory power of PCR MP (HGDI-0.939) is much higher than that of PFGE (HGDI-0.608). PFGE is an integral subtyping tool used by several national public health networks (e.g., PulseNet, FoodNet, and VetNet) to differentiate outbreak strain clusters [30]. On the other hand, *S.* Enteritidis is genetically highly monomorphic and PFGE-XbaI is known to display rather poor discriminatory potential for strains of this serovar [31]. PFGE-XbaI protocol lack discriminatory power to show the subtle genotypic differences that distinguish S. Enteritidis strains [32]. Here, the PFGE-E2 cluster with 32 isolates was separated into 11 clusters by PCR MP. Significantly, the number of clusters identified by PCR MP revealed the real epidemiological links among the isolates collected within a single farm (e.g., MP-E1, MP-E4) or those that originated from the same hatchery (e.g., MP-E2, MP-E4). Our findings demonstrated that analysis using PCR MP resulted in a more accurate picture of the phylogeny compared to PFGE. All clusters identified by PCR MP, but not by PFGE, carried strains belonging to the same serovar. Taking the above factors into account, we found PCR MP to be an intriguing one-step PCR-based method with high discriminatory power that may be useful, at least for the connection analysis of *S.* Enteritidis isolated from poultry. The discriminatory ability of this method is based on the gradual lowering of the denaturation temperature during PCR, which allows for the amplification of less stable DNA fragments (lower G + C content) and precludes the amplification of more stable fragments. As a result, we obtained characteristic DNA profiles that enabled the intraspecies genotyping of *Salmonella* strains. PCR MP proved a repeatable and specific method for differentiation of *Salmonella* strains, showing a discriminatory power high enough to identify the real epidemiological links between strains. PCR MP is not the ideal subtyping test as it meets six from the seven defined criteria including cost effectiveness, rapid performance, robust results, typeability, high discrimination and epidemiological concordance [33, 34]. Here the reproducibility was not demonstrated as PCR MP is sensitive to transferability. Based on the present results, PCR MP analysis seems a valuable adjunct to the methods based on sequencing. PCR MP might be an interesting secondary method to verify epidemiological links between strains identified by MLVA and/or MLST. However, to further confirm its usefulness in typing of various *Salmonella* serovars, its evaluation on a larger number of strains from various geographical regions would be necessary.

## Conclusions

PCR MP is highly discriminative, inexpensive, very fast method, which does not need a sophisticated equipment and demonstrates the usefulness in the epidemiological links analysis of a limited collection of *Salmonella* strains.
